# Inter, intra-examiner reliability and validity of inertial sensors to measure the active cervical range of motion in patients with primary headache

**DOI:** 10.17179/excli2021-3799

**Published:** 2021-05-10

**Authors:** Ignacio Elizagaray-García, Alfonso Gil-Martínez, Gonzalo Navarro-Fernández, Ana R. Navarro-Moreno, Jacqueline Sánchez-de-Toro-Hernández, Javier Díaz-de-Terán, Sergio Lerma-Lara

**Affiliations:** 1 Departamento de Fisioterapia, Centro Superior de Estudios Universitarios La Salle, Universidad Autónoma de Madrid. 28023 Madrid (Spain); 2 CranioSPain Research Group, Centro Superior de Estudios Universitarios La Salle, 28023 Madrid (Spain); 3 Motion in Brains Research Group, Centro Superior de Estudios Universitarios La Salle, 28023 Madrid (Spain); 4 Instituto de Rehabilitación Funcional y Ciencias Aplicadas al Deporte (IRF-La Salle), Centro Superior Estudios Universitarios La Salle, Madrid, Spain; 5 Instituto de Investigación Biosanitaria del Hospital Universitario La Paz, IdiPAZ, Madrid, Spain; 6 Unidad de Fisioterapia, Hospital Universitario La Paz, Madrid, Spain; 7 Departamento de Neurología, Hospital Universitario La Paz, Madrid, Spain

**Keywords:** primary headache, reliability, validity, physical examination, range of motion

## Abstract

We analyzed the inter- and intra-examiner reliability of Werium inertial sensors and the cervical range of motion (CROM) instrument for the measurement of active CROM (AcROM) in patients with primary headache. Another objective is to analyze the validity of the inertial sensors (Werium). The literature has reported symptomatology features in patients diagnosed with primary headache similar to that of patients with cervicogenic headache. The International Classification of Headache (ICHD-III) established the presence of reduced AcROM as a diagnostic criterion for cervicogenic headache. Several instruments are used for this measurement, with limitations in their applicability in daily clinical practice. A prospective longitudinal repeated measures study was conducted to assess the intra- and inter-rater reliability and validity of Werium inertial sensors in 20 adults with chronic primary headache. For the inter-rater analysis, the intraclass correlation coefficient (ICC) values were above 0.75 for all movements, indicating a good level of reliability. For the intra-rater results, the ICC values obtained by the Werium inertial sensors for all cervical movements were good for rater A (ICC >0.80) and rater B (ICC >0.84). For the validity, the ICCs obtained by the Werium inertial sensors compared with the CROM instrument for all cervical movements were moderate for both raters (ICC > 0.70, respectively). Values obtained in the standard error of measurement, minimum detectable change at 90% and limits of agreement also indicated good agreement. Werium inertial sensors have shown good to excellent reliability results, both intra- and inter-examiner (ICC > 0.75). Likewise, when the sensors were compared with another validated instrument (CROM device) they obtained high reliability results (ICC > 0.70). These results plus its relatively low price and ease of use allow us to recommend it in daily clinical practice to measure AcROM in patients with chronic primary headache.

## Abbreviations

CROM: Cervical range of motion

AcCROM: Active cervical range of motion

ICHD-III: International Classification of Headache

ICC: Intraclass Correlation coefficient

NDI: Neck Disability Index

HIT-6: Headache Impact Test

CIs: Confidence intervals

SEM: Standard error of measurement

MDC_90_: Minimum detectable change at 90 %

LOAs: Limits of agreement

## Introduction

Neck pain is a common symptom in patients with primary headache, such as migraine or tension-type headache (Ashina et al., 2015[4]). According to the current literature, however, despite this high prevalence, cervical musculoskeletal dysfunction appears not to play a role in the pathogenesis of primary headaches (Robertson and Morris, 2008[26]). Furthermore, the International Classification of Headache (ICHD-III) describes in point 11.2.1 a specific diagnostic subgroup (cervicogenic headache) to which it attributes cervical musculoskeletal dysfunction as being responsible for the headache. One of the diagnostic criteria described for this ICHD-III subgroup is the presence of reduced cervical range of motion (CROM) (Olesen, 2018[24]). According to one interesting study, most patients experiencing cervical pain underwent unnecessary treatments and medical exams; after an adequate evaluation, they presented typical migraine attacks without evidence of pathological conditions of the cervical spine (Viana et al., 2018[34])

Other symptomatology features, such as the location and the extent of the pain area and the presence of aura, photophobia, nausea and cervical musculoskeletal dysfunctions (e.g., forward head position, alterations in motor behavior or the presence of myofascial trigger points in the neck), appear to coexist in both patients with primary and cervicogenic headache (Elizagaray-Garcia et al., 2016[12]; Szikszay et al., 2018[31]; Liang et al., 2019[22]; Uthaikhup et al., 2020[32]). Therefore, the measurement of the active cervical range of motion (AcROM) is proposed as a necessary test to differentiate patients with cervicogenic headache from patients with primary headache.

Additionally, according to the literature, commonly used instruments for measuring AcROM include visual estimation, tape measurements, various types of goniometers and universal inclinometers, the CROM inclinometer and motion analysis using a 3D measurement system (Anoro-Hervera et al., 2019[3]). Along these lines, the gold standard for AcROM measurement is the 3D motion analysis laboratory, which allows measurement in terms of amplitude, velocity and quality, expressing these numerically and graphically. However, these specialized laboratories are inaccessible to the majority of healthcare professionals and are difficult to use in daily clinical practice due to their high cost, long time needed for measurement, and complexity (Jordan et al., 2000[17]; Anderst et al., 2011[1]).

Regarding AcROM measurement, there is no clear consensus on the most suitable instrument in the clinical setting; however, the CROM device is the most popular in daily practice (Solinger et al., 2000[29]; Assink et al., 2008[5]). This device was validated by Audette et al. in 2010, who reported high intra-examiner reliability (intraclass correlation coefficient [ICC] > 0.87) in both asymptomatic individuals and patients with neck pain (Fletcher and Bandy, 2008[13]; Audette et al., 2010[6]). However, this device presents the difficulty that the patient must maintain a static posture in the trunk while moving the neck, and the evaluator manually records the measured angle. Thus, if the patient does not keep the thoracic region stabilized, the CROM device could compensate for the cervical movement and it would provide erroneous data.

With the intention of overcoming these limitations, current technological advances are allowing the development of new digital instruments that improve AcROM assessment, such as portable inertial sensors. These inertial sensors have proven to be a faster, easier, and valid examination device for various parts of the human body. Also, their relatively inexpensive price makes them ideal tools for use in the examination of AcROM in daily clinical practice (Raya et al., 2018[25]; Costa et al., 2020[9]; Gobbo et al., 2020[14]).

These devices have been studied in various regions. Specifically, Werium Solutions inertial sensors have shown high intra- and inter-examination reliability in assessing elbow (ICC 0.83-0.96 and 0.94-0.97, respectively) and neck (ICC >0.70) movements (Raya et al., 2018[25]; Anoro-Hervera et al., 2019[3]; Costa et al., 2020[9]). Despite these results, however, no previous research has been performed in a symptomatic population such as patients with headache, nor have comparisons been made with another validated instrument commonly used in clinical practice, such as the CROM device.

Therefore, this study aimed to bring the results closer to daily clinical practice, where patients with headache are frequently evaluated. The main objective was to analyze the inter- and intra-examiner reliability of the Werium inertial sensors and the CROM instrument for the measurement of AcROM in patients with primary headache. Similarly, another objective of this study was to compare the measurement reliability between the 2 instruments (Werium and CROM).

## Methods

A prospective, longitudinal, repeated measures study was conducted to assess the intra- and inter-rater reliability of the Werium inertial sensors and the CROM inclinometer device. The validity of the Werium inertial sensors was also analyzed. The study was performed from January to September 2020 at La Paz University Hospital in Madrid (Spain). The protocol was approved by the Ethics Committee of La Paz University Hospital (code: PI-3442). All procedures followed the ethical standards of the declaration of Helsinki. All participants signed the informed consent document.

### Participants

All the participants were recruited using simple randomized sampling from the headache unit of the neurology department of La Paz University Hospital in Madrid (Spain). Patients were included if they met the following criteria: adults diagnosed with primary headache by a neurologist specialized in headache disorders according to ICHD-III (Olesen, 2018[24]). Individuals who were diagnosed with any type of headache other than primary headache were excluded, as well as those who had any type of musculoskeletal diagnosis, history of trauma or surgery in the cervico-cranio-mandibular region. We also excluded patients with history of drug abuse, meningitis, fibromyalgia, chronic pain in any region of the body, peripheral neuropathies, rheumatic diseases or other diseases with possible involvement of the sensory pathways. It should also be noted that due to ethical aspects, patients could continue their pharmacological treatment, although on the day of the evaluation they were required to be free of pain for the previous 6 hours.

### Variables and tools

#### Cervical range of motion

The AcROM of the neck was assessed by 2 experienced examiners with 2 different instruments (Werium inertial sensors and CROM instrument) and in 3 different planes (sagittal plane: flexion and extension; transversal plane: right and left rotations; frontal plane: right and left lateral flexions).

##### Inertial sensor (Werium Solutions)

The measurement equipment featured 2 inertial sensors (4 cm x 4 cm x 8 cm; weight < 200 g) (Werium Solutions, Madrid, Spain). Each sensor consists of a 3-axis accelerometer (ADXL 345 from Analog Devices) that analyzes the acceleration; a 3-axis gyroscope (ITG-3200 from Invense) that measures the angular velocity; and a 3-axis compass (HCM5883L from Honeywell) that assists the measurement by taking the Earth's magnetic field as a reference.

One sensor was attached with double-sided adhesive to the plastic front of the CROM instrument at the level of the forehead of each participant (mobile sensor), and the other sensor was attached to the anterior part of the thorax (at the level of the sternal manubrium). The measured angle was established by the relative angle between both sensors (Raya et al., 2018[25]). The sensors transmitted the signal via Bluetooth to the Pro Motion Capture software, which was installed on a Lenovo IdealPad 3 PC (AMD Ryzen 3-3250U). This Pro Motion Capture software executed the signal and calculated and displayed in real time the range of motion (ROM) of each of the participants' movements. This instrument has shown high intra-rater (ICC < 0.90) and inter-rater (ICC < 0.75) reliability (Raya et al., 2018[25]; Anoro-Hervera et al., 2019[3])

##### Cervical range of motion inclino-meter (CROM instrument)

The CROM instrument is a device that uses 2 inclinometers (frontal and lateral) for the evaluation of movements in the frontal and sagittal planes, respectively. In addition, it has the possibility of adapting a compass at the top of the instrument and a magnetic reference hanging around the participant's neck to measure rotational movements. Although this instrument is not the gold standard for measuring CROM, it is the most widely used clinically and has been extensively used in research, in which it is reported to have high inter- and intra-examiner reliability, (ICC=0.68-0.95) and (ICC=0.79-0.99), respectively (Audette et al., 2010[6]).

#### Neck Disability Index

The Neck Disability Index (NDI) is a self-administered questionnaire that determines neck pain and disability; it has 10 sections with 6 possible answers in each and is validated in Spanish (Andrade Ortega et al., 2010[2]). The score ranges from 10 to 60, which means that the higher the score, the greater the neck disability. The minimum detectable change is set at 5 points (Andrade Ortega et al., 2010[2]).

#### Headache Impact Test

The Headache Impact Test (HIT-6) is a self-administered questionnaire consisting of 6 items and 6 response options ("never"=6 items, "rarely"=8 items, "sometimes"=10 items, "very often"=11 items and "always"=13 items). It assesses the level of impact of the headache on the day-to-day life of each patient and is validated in Spanish (Martin et al., 2004[23]). The 4 headache impact severity categories are as follows: little or no impact (49 or less), some impact (50–55), substantial impact (56–59), and severe impact (60–78).

### Randomization

The evaluators measured the participants in each session following the GraphPad Software sequence for avoiding possible bias without any randomization of the examinators.

### Procedure

The research was performed in 2 visits to prevent possible bias in the results of the second measurement after numerous cervical movements in patients with high cervical disability (Liang et al., 2019[22]; Szikszay et al., 2019[30]; Elizagaray-Garcia et al., 2020[11]). During the first visit, each participant was provided with all the information related to the study and was given an additional information document to consult if required. Subsequently, the participant attended a question-and-answer session to consult for any relevant doubts, and the informed consent document was signed.

After all initial documentation was completed, demographic questions and self-administered questionnaires (NDI and HIT-6) were completed. Subsequently, the AcROM was assessed.

**Figure 1:**
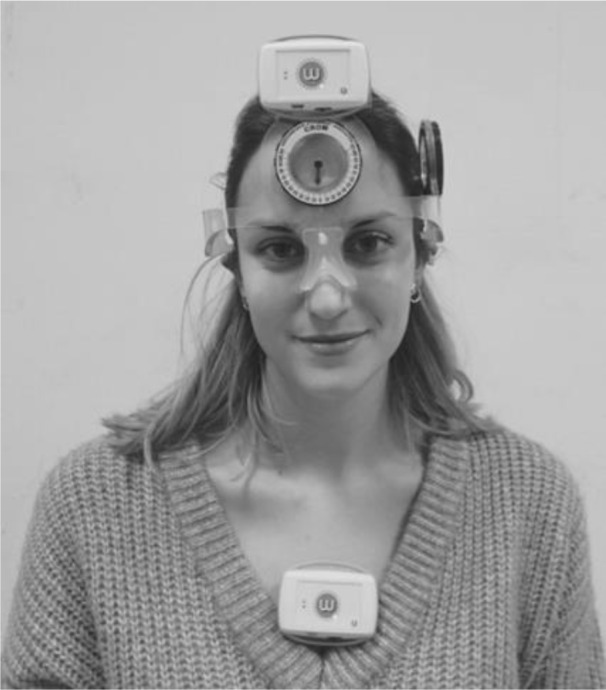
Simultaneous localization of the inertial sensors and the CROM instrument CROM, Cervical range of motion inclinometer

The first rater asked each participant to sit in a chair with their back against the backrest, feet flat on the floor, with ankles, knees and hips in 90° flexion and hands resting on the thighs. Subsequently, the CROM instrument was placed on the participants' heads according to the manufacturer's instructions, and both Werium sensors were placed in the regions mentioned above (Figure 1(Fig. 1)). Then, verbal commands were given to perform the movements of flexion, extension and lateral flexion (right and left) to the limit of each participant's capacity in each movement. Each participant had to repeat each movement twice consecutively.

Calibration of the sensors was performed only once at the beginning of the 4 movements in each plane, so that the results would not be altered by a different calibration each time. Simultaneous measurements from the inertial sensors and the CROM instrument were analyzed for flexion, extension and lateral flexion (right and left) movements. For the rotational movements, however, the movements made with the inertial sensors were measured first; then, in isolation (by removing the sensors), the rotations were recorded with the CROM. 

After the first tests, it was observed that when the rotations were measured simultaneously with the sensors and the CROM instrument, the compass contained in each inertial sensor was decalibrated due to its proximity to the magnetic reference of the CROM device, providing erroneous data. Therefore, we decided to simultaneously measure the movements of flexion, extension and lateral flexion (with the Werium and CROM device at the same time), then measure the rotations only with the sensors, and finally remove the sensors, place the CROM device’s magnetic reference and record the rotations with the CROM device.

**Figure 2:**
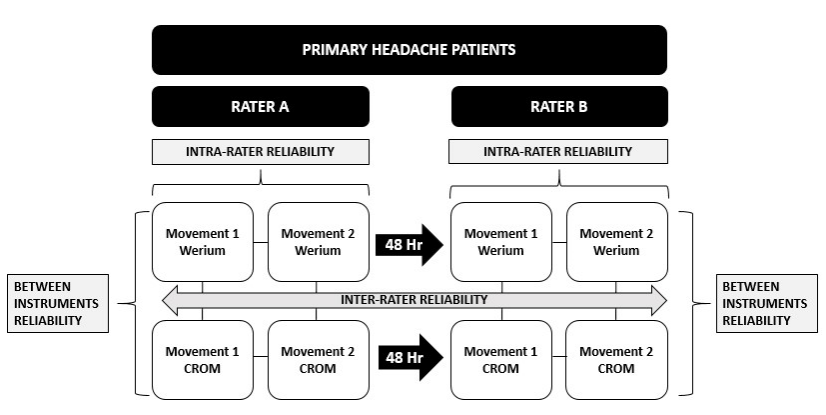
Procedure CROM, Cervical range of motion inclinometer

After the assessments, with the intention of ensuring a washout period for any symptoms generated by the measurements, a new appointment was arranged, respecting a minimum time of 48 hours between each of the appointments. In this second session, the measurements were performed in exactly the same manner, this time by a second rater (Figure 2(Fig. 2)).

### Sample size

The sample size was calculated using the ICC-based method (Donner and Eliasziw, 1987[10]; Walter et al., 1998[35]). Based on previous studies in which the ICCs of the CROM and Werium inertial sensors were respectively calculated, our ICC under the alternative hypothesis was estimated to be 0.90 (Fletcher and Bandy, 2008[13]; Raya et al., 2018[25]). A sample of at least 18 participants with 2 observations per participant was necessary to reach a statistical power of 80 % (ß=0.2) and to detect an ICC=0.90 when the ICC of the null hypothesis was 0.70, using an F-test with a significance level of 0.05. Taking into account possible dropouts from the study, we aimed to recruit at least 20 participants.

### Data analysis

The statistical analysis was performed with SPSS Statistics software (v.24.0; SPSS, Inc, Chicago, IL, USA). A total of 48 ICCs with 95 % confidence intervals (CIs) were calculated. For the inter-rater reliabilities, the averages of the 2 consecutive measures of each rater were used. Six ICC_(3,2)_s were performed per instrument, for a total of 12 ICC_(3,2)s_. For intra-examiner reliabilities, 6 ICC_(3,1)s_ were performed per rater and instrument, totaling 24 ICC_(3,1)s_, for which the first and second consecutive measurements of each movement were used. Finally, the validity (Werium inertial sensors vs CROM instrument) was analyzed, differentiating between rater A (6 ICC_[3,2]_) and rater B (6 ICC_[3,2]_), for which the averages of the 2 measurements of each movement were used (Weir, 2005[36]). ICC interpretations were made according to previously published categories to express levels of reliability: < 0.50 is poor agreement, 0.50-0.75 is moderate agreement, and > 0.75 is good to excellent agreement (Weir, 2005[36]).

The standard error of measurement (SEM) was calculated by the next mathematical formula, where RMS is the root mean squared total (Weir, 2005[36]). 


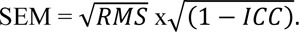


Responsiveness to change was analyzed using the minimum detectable change at 90 % (MDC_90_), and was calculated by the mathematical formula


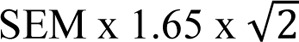


(Haley and Fragala-Pinkham, 2006[15]; Wyrwich, 2004[37]). The MDC_90 _expresses the minimum change necessary to be 90 % sure that the observed change between 2 measurements reflects a real change and not a measurement error.

Bland-Altman analysis was constructed by calculating the mean difference between 2 measurements (Weir, 2005[36]) Furthermore, the limits of agreement (LOAs) were determined as mean differences ± (standard deviation x 1.96) (Bland and Altman, 1995[7]; Bunce, 2009[8]). The calculation of the occurrence of systematic or random changes in the data was performed by calculating the 95 % CIs of the mean differences between the data values of 2 measurements. 

## Results

A total of 20 patients with primary headache were recruited for CROM analysis. Measurements were performed by 2 raters with extensive experience and on 2 different days. In addition, each rater performed the measurements with both tools (Werium and CROM). The demographic characteristics of the sample are listed in detail in Table 1(Tab. 1).

Inter-rater reliability descriptive statistics, ICCs, SEMs, MDC_90, _Bland-Altman analysis with 95 % CI, and LOA for each measurement are presented in Table 2(Tab. 2). All ICC values were above 0.75 for all movements with both (Werium and CROM), indicating a good level of reliability. Also, the range of MDC_90_ analyzed for inter-rater reliability was between 3.94 and 10.96 for the Werium and between 3.93 and 7.67 for the CROM device. In relation to SEMs, the values for all neck movements were SEM < 4.70 and < 3.29 for the Werium and the CROM device, respectively. 

Descriptive statistics of the intra-rater reliability, ICCs SEMs, MDC_90, _Bland-Altman analysis with 95 % CI, and LOA for each measurement made by each rater are presented in Tables 3(Tab. 3) and 4(Tab. 4). Both tables show a good ICC for all movements (ICC > 70) measured by rater A and rater B with both tools (Werium and CROM). Rater A also showed an MDC_90_ range from 5.61 to 11.41 for the Werium and from 5.63 and 8.69 for the CROM device (Table 3(Tab. 3)). Meanwhile, rater B reported an MDC_90_ range from 4.86 to 7.20 and from 5.80 to 8.44 for the measurements of the CROM device (Table 4(Tab. 4)). Regarding the SEM analysis, rater A showed a SEM < 4.89 and < 3.74 with the Werium and CROM device, respectively (Table 3(Tab. 3)). Meanwhile, rater B showed a SEM < 3.09 and < 3.62 with the Werium and CROM device, respectively (Table 4(Tab. 4)).

The validity analysis is presented in Table 5(Tab. 5). All ICC values were above 0.75 for rater A and above 0.69 for rater B. The range of MDC_90_ analyzed was between 5.15 and 10.33 for rater A and between 5.92 and 12.17 for rater B. Regarding the SEMs, the values for all neck movements were SEM < 4.43 and < 5.22 for rater A and rater B, respectively.

See also the Supplementary data.

## Discussion

The results of the present investigation show that the Werium sensors have moderate to excellent intra- and inter-examiner reliability, and validity for all movements (ICC > 0.70) except for left rotation made by rater B (ICC=0.70). According to the literature, ICC scores ranging from 0.75-0.90 are labelled as good, and those above 0.90 are described as excellent (Koo and Li, 2016[19]). Therefore, the results obtained in this study can be considered as good-excellent. 

According to the current evidence, four studies have analyzed the reliability of inertial sensors to assess CROM (Schiefer et al., 2015[27]; Raya et al., 2018[25]; Anoro-Hervera et al., 2019[3]; Gobbo et al., 2020[14]). All of them assessed inter-rater reliability (Raya et al., 2018[25]; Anoro-Hervera et al., 2019[3]; Gobbo et al., 2020[14]), two of them intra-rater reliability, (Schiefer et al., 2015[27]; Anoro-Hervera et al., 2019[3]), and two the validity of the inertial sensors (Schiefer et al., 2015[27]; Raya et al., 2018[25]). In this context, the inter-rater reliability results presented by the Werium inertial sensor in the present study (ICC > 0.75) are very similar to those presented by the inertial sensors of the four previous studies (Schiefer et al., 2015[27]; Raya et al., 2018[25]; Anoro-Hervera et al., 2019[3]; Gobbo et al., 2020[14]). However, only one of these analyzed the MDC_90_ and SEM, contributing more data to clinical practice (Anoro-Hervera et al., 2019[3]). In 2019, Anoro-Hervera et al. found good results, although poorer than those of our study, probably due to a different calibration methodology between each measurement. These authors calibrated the inertial sensors after each movement, whereas we calibrated only once before the first flexion, extension, rotation or lateral flexion movement. The difficulty of calibrating at the same neutral point for each repetition is likely to increase the chances of measurement error, given measurement could begin from different starting points.

In relation to intra-rater reliability, two studies to date have analyzed intra-rater reliability in inertial sensors (Schiefer et al., 2015[27]; Anoro-Hervera et al., 2019[3]). To this end, they used the mean of the standard deviation of five replicates, whereas Anoro-Hervera et al. (2019[3]), used the same methodology as ours, ICC_(3,1)._ However, our research was conducted with a symptomatic sample with moderate cervical disability and severe headache impact (Vernon and Mior, 1991[33]; Kosinski et al., 2003[20]), which brings the results closer to standard clinical practice. This fact could explain the lower repeatability of the intra-examiner results shown in Tables 2(Tab. 2) and 3(Tab. 3) with respect to those shown by Anoro-Hervera et al. (2019[3]). In addition, the Bland-Altman analysis shows negative values in the mean difference for all movements, which means that in the second movement there were higher ROMs than in the first. In this sense, we consider 2 possible hypotheses to explain it: (1) The fact of having performed the 2 movements consecutively could have provoked sufficient neuromusculoskeletal changes that could explain the greater AcROM recorded in all the second movements (Lascurain-Aguirrebena et al., 2016[21]); and (2) taking into account the Hawthorne effect, this could have triggered the participants' inherent need to improve their results in each of the second movements (Sedgwick and Greenwood, 2015[28]).

In addition, this study has a further clinical purpose. To increase internal validity in future clinical practice, the results obtained with the Werium sensors were compared with those recorded with an instrument (CROM instrument) that, although not the gold standard, has been validated. In this regard, two studies to date have analyzed the validation of inertial sensors (Schiefer et al., 2015[27]; Raya et al., 2018[25]). Raya et al. (2018[25]) analyzed the validation of the sensors by placing them in different positions and compared the results with the gold standard for motion analysis (3D camera system) in 8 asymptomatic individuals. Meanwhile, Schiefer et al. (2015[27]) performed a validation of the inertial sensors with respect to the normal values established in the literature. 

Again, the results of our research show a high ICC for rater A (ICC > 0.75) and rater B (ICC ≥ 0.70). It should be noted that rotational movements were not assessed by simultaneously placing the inertial sensors and the CROM instrument. Therefore, although the results were good-excellent, they should still be interpreted with caution because the rotational movements were not measured simultaneously.

### Clinical implications

According to the ICHD-III, reduced CROM is a diagnostic criterion for patients with cervicogenic headache and differentiates them from patients with other types of headache (Olesen, 2018[24]). With the current literature showing that patients with primary headache frequently present with neck pain, especially during the premonitory phase of migraine (Karsan et al., 2018[18]) and that similar symptomatology features in patients with cervicogenic headache, the results of this study suggest that the Werium inertial sensors could be reliable instruments and could facilitate the examination of clinicians treating patients with headache. In addition, having at disposal cheap digital instruments to objectively evaluate patients with headache would be a crystalline clinical improvement that would prevent unnecessary medical tests (including radiation exposure) and treatments. Lastly, the smaller size of the clinical instruments used might be important, and in this case the difference is considerable.

### Limitations

This study has several limitations. First, the intra-examiner reliability analysis was performed as single consecutive values. Future studies should address this limitation by considering several measurements at different times, or at least to ensure that there is enough time between each consecutive measurement to ensure that the second measurement is not affected by neuromuscular changes of the first. Second, the reliability between devices for the rotation movement must be interpreted with caution because they were not measured simultaneously due to the incompatibility of the inertial sensors with the magnetic reference of the CROM instrument. In this sense, rotational movement has particular relevance in patients with headache due to the limitation observed in patients with cervicogenic headache (Hall et al., 2008[16]). Another limitation is that although the results of this study are reliable, we cannot say categorically that the inertial sensors are a valid instrument to measure AcROM in patients with headache because it should have been compared with the gold standard device and not with another validated instrument, such as the CROM inclinometer. Also, in our Bland-Altman analysis, we have obtained some statistically significative mean differences. Even though those differences were all lower than the MDC_90_, authors recommend to interpret those results with caution. Finally, recent systematic reviews with meta-analyses indicate that patients with chronic primary headache present higher forward head posture than those with episodic primary headache and asymptomatic individuals (Elizagaray-Garcia et al., 2020[11]); thus, we consider it a limitation that the sensor does not measure this type of movement and we propose it as a future line of research.

## Conclusion

The results obtained in this study of the Werium inertial sensors suggest that it is a good, reliable instrument with moderate to excellent intra- and inter-examiner ICC values (ICC ≥ 0.71). Similarly, it has been shown that when compared with another validated instrument (CROM device) it has moderate to excellent reliability for all movements (ICC ≥ 0.70). Thus, the results obtained plus its low relative price and ease of use allow us to recommend the use of these sensors in daily clinical practice to measure AcROM in patients with primary headache. 

## Conflict of interest

Dr. Sergio Lerma-Lara declares that he has commercial relationships with Werium Solutions. The rest of the authors declare they have no conflicts of interest.

## Financial support

This research received no external funding.

## Acknowledgement

We thank the Centro Superior de Estudios Universitarios La Salle for its support in financing the professional English editing of the manuscript.

## Supplementary Material

Supplementary data

## Figures and Tables

**Table 1:**
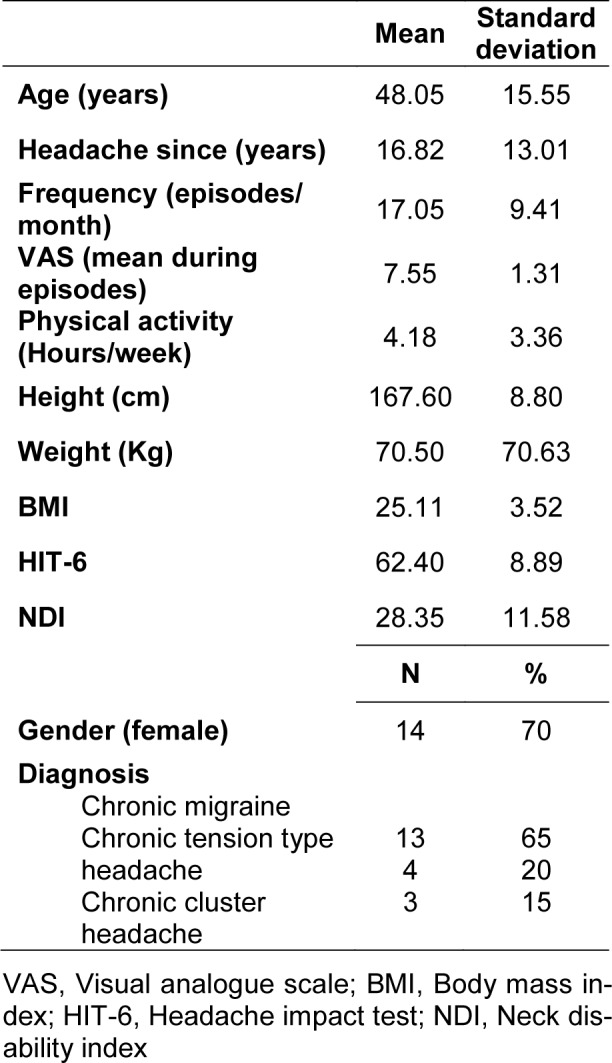
Demographic characteristics of the sample

**Table 2:**
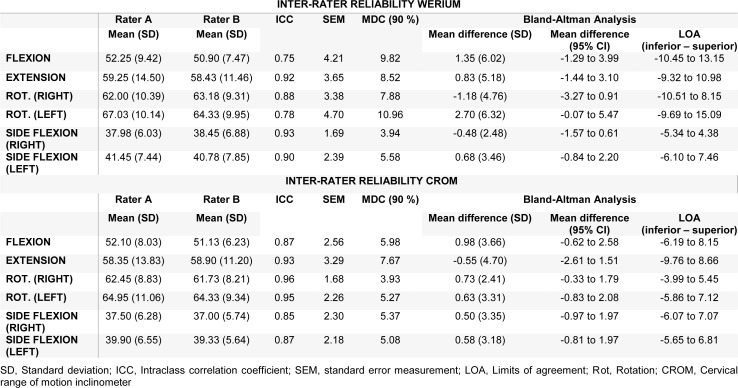
Inter-rater reliability of Werium and CROM

**Table 3:**
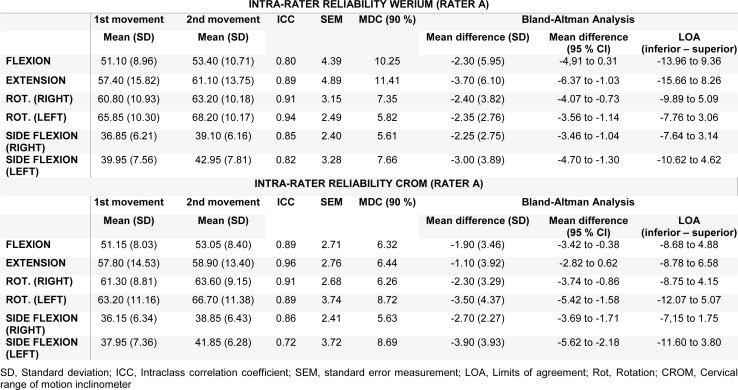
Intra-rater reliability. Rater A with Werium and CROM

**Table 4:**
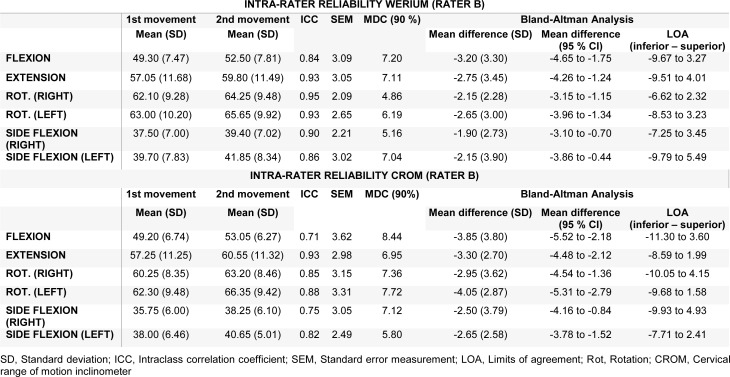
Intra-rater reliability. Rater B with Werium and CROM

**Table 5:**
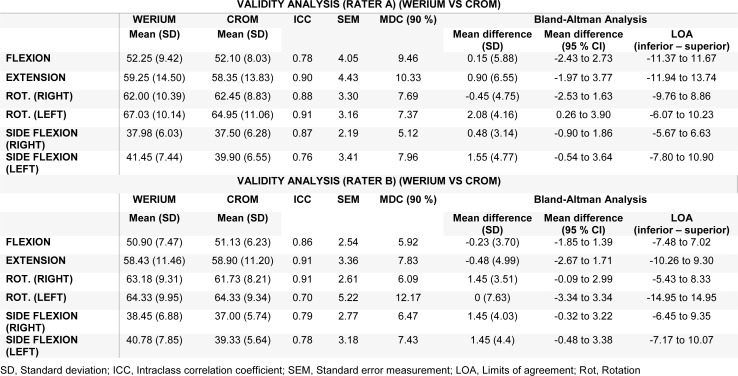
Validity analysis (Werium vs CROM)

## References

[1] Anderst WJ, Baillargeon E, Donaldson WF, Lee JY, Kang JD (2011). Validation of a noninvasive technique to precisely measure in vivo three-dimensional cervical spine movement. Spine.

[2] Andrade Ortega JA, Delgado Martínez AD, Ruiz RA (2010). Validation of the Spanish Version of the Neck Disability Index. Spine.

[3] Anoro-Hervera A, Lafuente-Pérez A, Navarro-Fernández G, Muñoz-García D, Lerma-Lara S, Beltran-Alacreu H (2019). Intra-rater and inter-rater reliability of cervical active range of movement in young asymptomatic adults using inertial sensors. Expert Rev Med Devices.

[4] Ashina S, Bendtsen L, Lyngberg AC, Lipton RB, Hajiyeva N, Jensen R (2015). Prevalence of neck pain in migraine and tension-type headache: A population study. Cephalalgia.

[5] Assink N, Bergman GJD, Knoester B, Winters JC, Dijkstra PU (2008). Assessment of the cervical range of motion over time, differences between results of the Flock of Birds and the EDI-320: A comparison between an electromagnetic tracking system and an electronic inclinometer. Man Ther. Man Ther.

[6] Audette I, Dumas JP, Côté JN, De Serres SJ (2010). Validity and between-day reliability of the cervical range of motion (CROM) device. J Orthop Sports Phys Ther.

[7] Bland JM, Altman DG (1995). Comparing methods of measurement: Why plotting difference against standard method is misleading. Lancet.

[8] Bunce C (2009). Correlation, agreement, and Bland-Altman analysis: Statistical analysis of method comparison studies. Am J Ophthalmol.

[9] Costa V, Ramírez Ó, Otero A, Muñoz-García D, Uribarri S, Raya R (2020). Validity and reliability of inertial sensors for elbow and wrist range of motion assessment. PeerJ.

[10] Donner A, Eliasziw M (1987). Sample size requirements for reliability studies. Stat Med.

[11] Elizagaray-Garcia I, Beltran-Alacreu H, Angulo-Díaz S, Garrigós-Pedrón M, Gil-Martínez A (2020). Chronic Primary headache subjects have greater forward head posture than asymptomatic and episodic primary headache sufferers: Systematic review and meta-analysis. Pain Med.

[12] Elizagaray-Garcia I, Muriente-Gonzalez J, Gil-Martinez A (2016). Education for patients with fibromyalgia. A systematic review of randomised clinical trials. Rev Neurol.

[13] Fletcher JP, Bandy WD (2008). Intrarater reliability of CROM measurement of cervical spine active range of motion in persons with and without neck pain. J Orthop Sports Phys Ther.

[14] Gobbo S, Vendramin B, Roma E, Duregon F, Bocalini DS, Rica RL (2020). Reliability of an integrated inertial sensor for the continuous measurement of active cervical range of motion in a group of younger and elderly individuals. J Funct Morphol Kinesiol.

[15] Haley SM, Fragala-Pinkham MA (2006). Interpreting change scores of tests and measures used in physical therapy. Phys Ther.

[16] Hall T, Briffa K, Hopper D (2008). Clinical evaluation of cervicogenic headache: A clinical perspective. J Man Manip Ther.

[17] Jordan K, Dziedzic K, Jones PW, Ong BN, Dawes PT (2000). The reliability of the three-dimensional FASTRAK measurement system in measuring cervical spine and shoulder range of motion in healthy subjects. Rheumatology.

[18] Karsan N, Bose P, Goadsby PJ (2018). The migraine premonitory phase. Continuum (Minneapolis).

[19] Koo TK, Li MY (2016). A guideline of selecting and reporting intraclass correlation coefficients for reliability research. J Chiropr Med.

[20] Kosinski M, Bayliss MS, Bjorner JB, Ware JE, Garber WH, Batenhorst A (2003). A six-item short-form survey for measuring headache impact: The HIT-6TM. Qual Life Res.

[21] Lascurain-Aguirrebena I, Newham D, Critchley DJ (2016). Mechanism of action of spinal mobilizations a systematic review. Spine.

[22] Liang Z, Galea O, Thomas L, Jull G, Treleaven J (2019). Cervical musculoskeletal impairments in migraine and tension type headache: A systematic review and meta-analysis. Musculoskelet Sci Pract.

[23] Martin M, Blaisdell B, Kwong JW, Bjorner JB (2004). The Short-Form Headache Impact Test (HIT-6) was psychometrically equivalent in nine languages. J Clin Epidemiol.

[24] Olesen J (2018). Headache Classification Committee of the International Headache Society (IHS). The International Classification of Headache Disorders, 3rd ed. Cephalalgia.

[25] Raya R, Garcia-Carmona R, Sanchez C, Urendes E, Ramirez O, Martin A (2018). An inexpensive and easy to use cervical range of motion measurement solution using inertial sensors. Sensors.

[26] Robertson B, Morris M (2008). The role of cervical dysfunction in migraine: a systematic review. Cephalalgia.

[27] Schiefer C, Kraus T, Ellegast RP, Ochsmann E (2015). A technical support tool for joint range of motion determination in functional diagnostics-an inter-rater study. J Occup Med Toxicol.

[28] Sedgwick P, Greenwood N (2015). Understanding the hawthorne effect. BMJ.

[29] Solinger AB, Chen J, Lantz CA (2000). Standardized initial head position in cervical range-of-motion assessment: Reliability and error analysis. J Manipulative Physiol Ther.

[30] Szikszay TM, Hoenick S, von Korn K, Meise R, Schwarz A, Starke W (2019). Which examination tests detect differences in cervical musculoskeletal impairments in people with migraine? A systematic review and meta-analysis. Phys Ther.

[31] Szikszay TM, Luedtke K, von Piekartz H (2018). Increased mechanosensivity of the greater occipital nerve in subjects with side-dominant head and neck pain - a diagnostic case-control study. J Man Manip Ther.

[32] Uthaikhup S, Barbero M, Falla D, Sremakaew M, Tanrprawate S, Nudsasarn A (2020). Profiling the extent and location of pain in migraine and cervicogenic headache: A cross-sectional single-site observational study. Pain Med.

[33] Vernon H, Mior S (1991). The neck disability index: A study of reliability and validity. J Manipulative Physiol Ther.

[34] Viana M, Sances G, Terrazzino S, Sprenger T, Nappi G, Tassorelli C (2018). When cervical pain is actually migraine: An observational study in 207 patients. Cephalalgia.

[35] Walter SD, Eliasziw M, Donner A (1998). Sample size and optimal designs for reliability studies. Stat Med.

[36] Weir JP (2005). Quantifying test-retest reliability using the intraclass correlation coeffcient and the SEM. J Strength Cond Res.

[37] Wyrwich KW (2004). Minimal important difference thresholds and the standard error of measurement: is there a connection?. J Biopharm Stat.

